# Adiponectin Is Related to Cardiovascular Risk in Severe Mental Illness Independent of Antipsychotic Treatment

**DOI:** 10.3389/fpsyt.2021.623192

**Published:** 2021-05-28

**Authors:** Elina J. Reponen, Martin Tesli, Ingrid Dieset, Nils Eiel Steen, Trude S. J. Vedal, Attila Szabo, Maren C. F. Werner, Synve H. Lunding, Ingrid T. Johansen, Linn N. Rødevand, Ole A. Andreassen, Thor Ueland

**Affiliations:** ^1^NORMENT, Norwegian Centre for Mental Disorders Research, Oslo University Hospital and University of Oslo, Oslo, Norway; ^2^Department of Mental Disorders, Norwegian Institute of Public Health, Oslo, Norway; ^3^Division of Mental Health and Addiction, Acute Psychiatric Department, Oslo University Hospital, Oslo, Norway; ^4^Institute of Clinical Medicine, University of Oslo, Oslo, Norway; ^5^Research Institute of Internal Medicine, Oslo University Hospital Rikshospitalet, Oslo, Norway; ^6^K.G. Jebsen Thrombosis Research and Expertise Center, University of Tromsø, Tromsø, Norway

**Keywords:** leptin, adiponectin, L/A ratio, CVD risk, antipsychotic treatment, schizophrenia, bipolar disorder

## Abstract

**Background:** Schizophrenia (SCZ) and bipolar disorder (BD) are severe mental illnesses (SMI) associated with elevated cardiovascular disease (CVD) risk, including obesity. Leptin and adiponectin are secreted by adipose tissue, with pro- and anti-inflammatory properties, respectively. The second generation antipsychotics (AP) olanzapine, clozapine, and quetiapine have been associated with high leptin levels in SMI. However, the link between inflammatory dysregulation of leptin and adiponectin and CVD risk in SMI, and how this risk is influenced by body mass and AP medication, is still not completely understood. We investigated herein if leptin, adiponectin or their ratio (L/A ratio) could predict increased CVD risk in SCZ, BD, and in subgroups according to use of antipsychotic (AP) treatment, independent of other cardio-metabolic risk factors.

**Methods:** We measured fasting plasma levels of leptin and adiponectin, and calculated the L/A ratio in *n* = 1,092 patients with SCZ and BD, in subgroups according to AP treatment, and in *n* = 176 healthy controls (HC). Differences in the levels of adipokines and L/A between groups were examined in multivariate analysis of covariance, and the correlations between adipokines and body mass index (BMI) with linear regression. CVD risk was defined by total cholesterol/high-density lipoprotein (TC/HDL) and triglyceride/HDL (TG/HDL) ratios. The adipokines and L/A ratios ability to discriminate individuals with TG/HDL and TC/HDL ratios above threshold levels was explored by ROC analysis, and we investigated the possible influence of other cardio-metabolic risk factors on the association in logistic regression analyses.

**Results:** We observed higher leptin levels and L/A ratios in SMI compared with HC but found no differences in adiponectin. Both adipokines were highly correlated with BMI. The low adiponectin levels showed a fair discrimination in ROC analysis of individuals with CVD risk, with AUC between 0.7 and 0.8 for both TC/HDL and TG/HDL, in all groups examined regardless of diagnosis or AP treatment. Adiponectin remained significantly associated with an elevated TC/HDL and TG/HDL ratio in SMI, also after further adjustment with other cardio-metabolic risk factors.

**Conclusions:** Adiponectin is not dysregulated in SMI but is associated with CVD risk regardless of AP treatment regime.

## Introduction

Schizophrenia (SCZ) and bipolar disorder (BD) are severe mental illnesses (SMI) that are associated with an increased cardiovascular disease (CVD) risk (1). Obesity occurs frequently in patients with SCZ and BD and contributes to the elevated cardiovascular risk (2). The prevalence of elevated body-mass index (BMI) in patients with SCZ and BD is estimated to be 3–5 times higher compared with the general population (2, 3).

A pro-inflammatory state of the adipose tissue is supposed to accelerate CVD in obese and overweighed subjects and is characterized by augmented production of inflammatory cytokines (4). Thus, leptin and adiponectin are cytokines primarily secreted by adipose tissue (adipokines) with pro- and anti-inflammatory properties, respectively (5). These proteins may have direct effects on atherogenesis in CVD, and experimental studies have demonstrated that leptin promotes, whereas adiponectin attenuates atherosclerosis (6), although a dual role on endothelial cells has recently been described for adiponectin (7). Furthermore, numerous clinical studies implicate dysregulated leptin and adiponectin levels in the progression of CVD (8–11). Thus, both hyperleptinemia and hypoadiponectinemia have been shown to be independently associated with increased fat tissue and CVD risk in the general population. Due to the opposite metabolic effects of leptin and adiponectin, the leptin/adiponectin ratio (L/A ratio) has been proposed as a useful marker for metabolic disease, and may be more strongly associated with CVD risk than leptin or adiponectin alone (12, 13).

Schizophrenia and related disorders are associated with dysregulated adipokine levels (14) and we and other have demonstrated that use of antipsychotic (AP) medication (15), especially the 3 s generation antipsychotics olanzapine, clozapine, and quetiapine (16, 17) may enhance the leptin as well as the L/A ratio (15) in SMI. However, the link between dysregulation of the leptin-adiponectin axis and CVD in SMI, and how this risk is modified by body mass and AP medication, have scarcely been examined. Herein vi investigate whether leptin, adiponectin or their ratio (L/A ratio) could be associated with increased CVD risk in SCZ and BD, and in subgroups according to AP treatment, independent of other established cardio-metabolic risk factors.

Our specific aims of this study were 4-fold. Firstly, we evaluate whether the distribution of leptin and adiponectin and their ratio differ between patients with SMI (SCZ or BD) compared to healthy controls (HC), and between subgroups of AP medication, and secondly if this difference is mitigated by BMI. Thirdly, we investigate if these adipokines or their ratio can discriminate individuals with or without pro-atherogenic lipid ratios above threshold levels, and fourthly, if any association with these lipid ratios is independent or modified by other established cardio-metabolic risk factors: age, sex, BMI, C-reactive protein (CRP), insulin resistance (HOMA-IR), smoking, and anti-psychotic, anticonvulsant, and lithium treatment dose (DDD). As a measure of CVD risk we calculated pro-atherogenic lipid ratios total cholesterol/high-density lipoprotein; HDL-c (TC/HDL) and triglyceride/HDL-c (TG/HDL), based on our previously published results (18).

We hypothesize to find significantly higher leptin levels and significantly lower adiponectin levels in SCZ and BD compared to healthy controls, mitigated by BMI. We expect the dysregulated adipokine levels and particularly L/A to independently predict elevated atherogenic lipid ratios in all groups, and in particular in patients using AP treatment, and in patients using olanzapine, clozapine or quetiapine.

## Methods

### Design and Ethics

This cross-sectional study is a part of the large ongoing Thematically Organized Psychosis (TOP) Study at the Norwegian Centre for Mental Disorders Research (NORMENT). Patients in the TOP study are included from hospitals and outpatient clinics in the Oslo, Trondheim, and Lillehammer regions in Norway. The sample for this current study consists of patients and healthy controls included from year 2002 until 2015, all with fasting blood samples available. Both the patients and the HC have given written informed consent, and the study was approved by the Norwegian Scientific Ethical Committees and the Norwegian Data Protection Agency.

### Sample

#### Patients

In the current study, 701 patients with schizophrenia spectrum disorder and 391 patients with bipolar spectrum disorder were included, with age between 18 and 65 years.

The diagnostic evaluation of the patients was based on the SCID-1 (Structured Clinical Interview in Diagnostic and Statistical Manual of Mental Disorders, 4th Edition (DSM-IV) axis I Disorders) [The inter-investigator diagnostic agreement has previously been evaluated to a satisfying level of 82%, with overall κ = 0.77 (CI 0.60–0.94) (19)]. Symptoms were evaluated using the Positive and Negative Syndrome Scale (PANSS), and the Calgary Depression Scale for Schizophrenia (CDSS), and patient medication records and smoking habits were registered.

A diagnosis of schizophrenia spectrum disorder (SCZ) included the diagnoses of schizophrenia, schizoaffective disorder, schizophreniform disorder, and psychotic disorder not otherwise specified, while a diagnosis of bipolar spectrum disorder (BD) included the diagnoses of bipolar I, bipolar II, and bipolar disorder not otherwise specified.

In this study, SMI is defined as the SCZ and BD groups combined. The subgroups according to antipsychotic treatment were defined as follows: those patients with SMI receiving second generation AP treatment with olanzapine, clozapine or quetiapine were the AP^O/C/Q^ (*n* = 522) group, those receiving other, first or second generation, AP treatment were the AP (*n* = 269) group, and those patients who did not receive antipsychotic treatment were the AP- (*n* = 301) group.

#### Healthy Controls

The current study included 176 healthy controls (HC), between 18 and 65 years old. The HC were randomly selected from statistical records (www.ssb.no) in the Oslo region. Exclusion criterion for HC was current or previous SMI in index persons or their family members, assessed with the clinical interview Primary Care Evaluation of Mental Disorders (PRIME MD).

### The Exclusion Criteria

The exclusion criteria for all participants in the study were: on-going infections, C-reactive protein (hs-CRP) >20 mg/L of any reason, on-going autoimmune or inflammatory diseases, on-going cancer, treatment with immune modulating medication of any reason, or insulin levels <400 pmol/L (for valid calculation of insulin resistance). As we investigated atherogenic lipid ratios in our study, a diabetic profile or other dysregulated metabolic parameters were not an exclusion criteria.

### Body Mass Index

All participants were weighed on calibrated digital weights under standard conditions, height was measured with standard methods and body mass index (BMI) (kg/m^2^) calculated.

### Defined Daily Dose for Antipsychotics, Anticonvulsants, and Lithium

Information on the use of prescribed antipsychotics, anticonvulsants, and lithium was assessed by clinical interview and hospital records. “Defined daily dose” (DDD) of the medication was calculated according to the World Health Organization (WHO) principles. For antipsychotic or anticonvulsant medication, we calculated the individual total DDD based on polypharmacy. The DDD is the assumed average maintenance dose per day for a drug used for its main indication in adults and provide a fixed unit of measurement independent of dosage form (http://www.whocc.no/atc_ddd_index/).

### Blood Samples

Fasting blood samples were collected between 8 am and 11 am for the most participants. Blood samples were drawn into EDTA tubes, stored at room temperature for 45 min and placed in refrigerator at 4 degrees C. They were then transported to the Biobank the following workday, where 2 x 9 ml EDTA tubes were centrifuged at 1,800 g for 15 min. Plasma was collected and stored at −80 degrees C in multiple aliquots (20).

#### Biochemistry

Plasma levels of total cholesterol (TC), triglyceride (TG), high-density lipoprotein (HDL-c), and low-density lipoprotein (LDL-c) were measured on an Integra 800 instrument from Roche Diagnostics, according to standard methods. Leptin, adiponectin and C-reactive protein (CRP) were analyzed using standardized platforms from Roche Diagnostics. All analyses were performed at the Department of Medical Biochemistry, Oslo University Hospital.

#### Insulin Resistance

Glucose and insulin were analyzed at the Department of Medical Biochemistry, Oslo University Hospital. Glucose levels were analyzed using standardized platforms from Roche Diagnostics. Insulin was analyzed at the Hormone Laboratory by radioimmunoassay (RIA) using standard methods. We estimated insulin resistance using the Homeostasis Model Assessment for Insulin Resistance (HOMA-IR) (21). As the calculation is valid only with insulin levels <400 pmol/L, participants with higher levels were excluded (*n* = 11).

#### Cardiovascular Risk

Cardiovascular risk was estimated calculating established pro-atherogenic lipid ratios including TG/HDL and TC/HDL, with sex-dependent cut-offs established elsewhere (22, 23).

### Statistical Analyses

All statistical analyses were done using the SPSS software package for Windows, version 26.0 (SPSS Chicago. USA). All analyses were two-tailed with a level of significance set at *p* < 0.05. All skewed data was log-transformed prior to further analyses. Demographics of the study population were analyzed with analysis of covariance (ANOVA) for continuous variables and chi-square test for independence for categorical variables.

Differences in levels of adipokines, between HC and diagnostic groups (i.e., SCZ and BD) were analyzed by multivariate analysis of covariance (MANCOVA), adjusting stepwise for age, sex, duration of illness, and BMI. The same analysis model was then repeated for the AP, AP-, and AP^O/C/Q^ subgroups. Further adjustment included mood stabilizers; anticonvulsive, and lithium treatment dose (DDD) as well as duration of AP treatment. We then analyzed correlations with linear regression analysis between BMI and leptin, adiponectin or L/A ratio across the studied groups.

To evaluate if adipokines or their ratio could discriminate individuals with or without pro-atherogenic lipid ratios (TG/HDL and TC/HDL) above threshold levels, we then performed receiver operating characteristics (ROC) analysis of leptin, adiponectin, and L/A ratio.

Finally, we evaluated the association between adipokine levels and pro-atherogenic risk using logistic regression with different adjustment levels to assess if the associations were independent or modified by other cardio-metabolic risk factors in BD, SCZ and HC. The same analysis model was then repeated for the AP, AP-, and AP^O/C/Q^ subgroups. Multivariable adjustment included age, sex, BMI, C-reactive protein (CRP), insulin resistance (HOMA-IR), smoking, anti-psychotic, anticonvulsive, and lithium treatment dose (DDD), duration of AP treatment and duration of illness.

## Results

### Sample Characteristics

The clinical characteristics of the study population according to AP treatment are shown in Table 1 and the clinical characteristics according to diagnostic groups are shown in Supplementary Table 1. Patients with SMI were of a similar age compared to HC, but when looking at the diagnostic groups, patients with BD were older than SCZ and had a longer duration of illness. Patients were less frequently male and of European origin than HC. Patients with SCZ received more often anti-psychotic treatment than BD but less anticonvulsants and lithium. Comparing a range of cardio-metabolic risk factors including HOMA-IR, CRP, BMI and lipids revealed a generally higher burden in patients with SMI was revealed, especially in SCZ compared with HC. Focusing on pro-atherogenic lipid ratios, patients with SMI had a 2- and 3-times higher proportion of individuals with TC/HDL and TG/HDL above threshold limits, respectively. Evaluated within the diagnostic groups, particularly SCZ patients had elevated ratios. These findings are similar to our previously published results on pro-atherogenic lipid ratios in a partly overlapping sample (18).

**Table 1:** Demographics of the study population.

	**HC**	**SMI**	**AP-**	**AP**	**APO/C/Q**	***Post-hoc***
N	176	1092	301	269	522	
Sex (male)	113 (64)	576 (53)^**^	139 (46)	141 (52)	296 (57)	HC, APO/C/Q>AP-
Age	32 ± 8	32 ± 11	33 ± 12	31 ± 10	31 ± 10	
Ethnicity (European)	173 (98)	893 (82)^***^	254 (84)	209 (78)	430 (82)	HC>AP-,AP,APO/C/Q
Duration of illness, years	N/A	9.9 (8.9)	12.0 (9.8)	8.9 (8.1)	9.1 (8.5)	AP-> AP,APO/C/Q
Daily smoking	N/A	492 (46)	120 (41)	135 (52)	237 (46)	
Statin use	0 (0)	15 (1.5)	1 (0.3)	9 (3.3)	6 (1.1)	AP>AP-
Antipsychotics (DDD)	N/A	0.88 (0.96)	0 (0)	0.93 (0.69)	1.35 (1.01)	APO/C/Q>AP>AP-
Duration of AP treatment, months	N/A	9.7 (24.6)	0 (0)	11.6 (29.4)	14.2 (27.1)	
Anticonvulsants (DDD)	N/A	0.14 (0.35)	0.17 (0.39)	0.10 (0.29)	0.14 (0.35)	AP-> AP
Lithium (DDD)	N/A	0.08 (0.30)	0.09 (0.33)	0.05 (0.22)	0.09 (0.32)	APO/C/Q>AP
**Cardiometabolic Risk Factors**						
HOMA-IR	1.2 ± 0.7	1.7 ± 1.0^***^	1.4 ± 0.8	1.7 ± 0.9	1.8 ± 1.1	APO/C/Q,AP>AP-,HC
CRP (mg/L)	1.6 ± 2.2	2.3 ± 2.8^***^	2.0 ± 2.5	2.6 ± 3.3	2.4 ± 2.7	APO/C/Q,AP>AP->HC
BMI	24.2 ± 3.6	25.8 ± 4.6^***^	24.8 ± 4.3	26.3 ± 5.0	26.0 ± 4.5	APO/C/Q,AP>AP-,HC
HDL-c(mmol/L)	1.47 ± 0.39	1.38 ± 0.43^*^	1.48 ± 0.45	1.35 ± 0.41	1.34 ± 0.42	HC,AP->AP,APO/C/Q
LDL-c (mmol/L)	2.94 ± 0.88	3.13 ± 0.95^*^	3.01 ± 0.90	3.05 ± 0.89	3.24 ± 0.99	APO/C/Q>HC,AP-,AP
Total-c (mmol/L)	4.70 ± 0.95	5.07 ± 1.07^***^	4.97 ± 1.07	5.03 (1.02)	5.16 ± 1.09	AP-,AP,APO/C/Q>HC
Triglycerides (mmol/L)	1.07 ± 0.78	1.39 ± 0.99^***^	1.19 ± 0.71	1.48 ± 1.19	1.45 ± 1.00	AP,APO/C/Q>HC,AP-
Total-c/HDL-c	21 (12)	257 (24)	50 (17)	65 (24)	142 (27)	APO/C/Q,AP>AP->HC
Triglycerides/HDL-c	17 (10)	301 (28)	58 (19)	85 (32)	158 (30)	APO/C/Q,AP>AP->HC
**Symptom scores**						
PANSS total	N/A	0.22 ± 0.13	0.22 ± 0.12	0.24 ± 0.12	0.22 ± 0.13	
CDSS total	N/A	5.3 ± 4.7	5.7 ± 4.4	5.5 ± 5.2	5.0 ± 4.6	

*Analyzed with ANOVA for continuous variables and chi-square test for categorical variables. HC, healthy controls; SMI, severe mental illness; AP-, patients not using antipsychotic treatment; APO/C/Q, patients using olanzapine, clozapine or quetiapine; AP, patients using other AP; n, number; DDD, defined daily dose; HOMA-IR, homeostasis model assessment for insulin resistance; CRP, C-reactive protein; mg/L, milligrams per liter; mmol/L, millimoles per liter; BMI, body mass index; HDL-c, high density lipoprotein cholesterol; LDL-c, low density lipoprotein cholesterol; Total-c, total cholesterol; PANSS, Positive and Negative Syndrome Scale; CDSS, Calgary Depression Scale for Schizophrenia; N/A, not applicable; n.s., not significant*,

* *p < 0.05*

** *p < 0.01*

*** *p < 0.001 vs. HC*.

Evaluation of demographics according to antipsychotic (AP) treatment revealed a lower proportion of male patients that did not receive AP treatment (AP-). In general, patients using AP (including AP^O/C/Q^) had a higher metabolic burden compared with those not receiving AP (both AP- and HC). The highest proportion of dysregulated pro-atherogenic lipid ratios (i.e., TC/HDL and TG/HDL) were observed in patients using AP (including AP^O/C/Q^) followed by those not using AP (AP-), and then HC.

### Adipokine and L/A Ratio Levels in SMI and Between AP Subgroups

**Figure 1:**
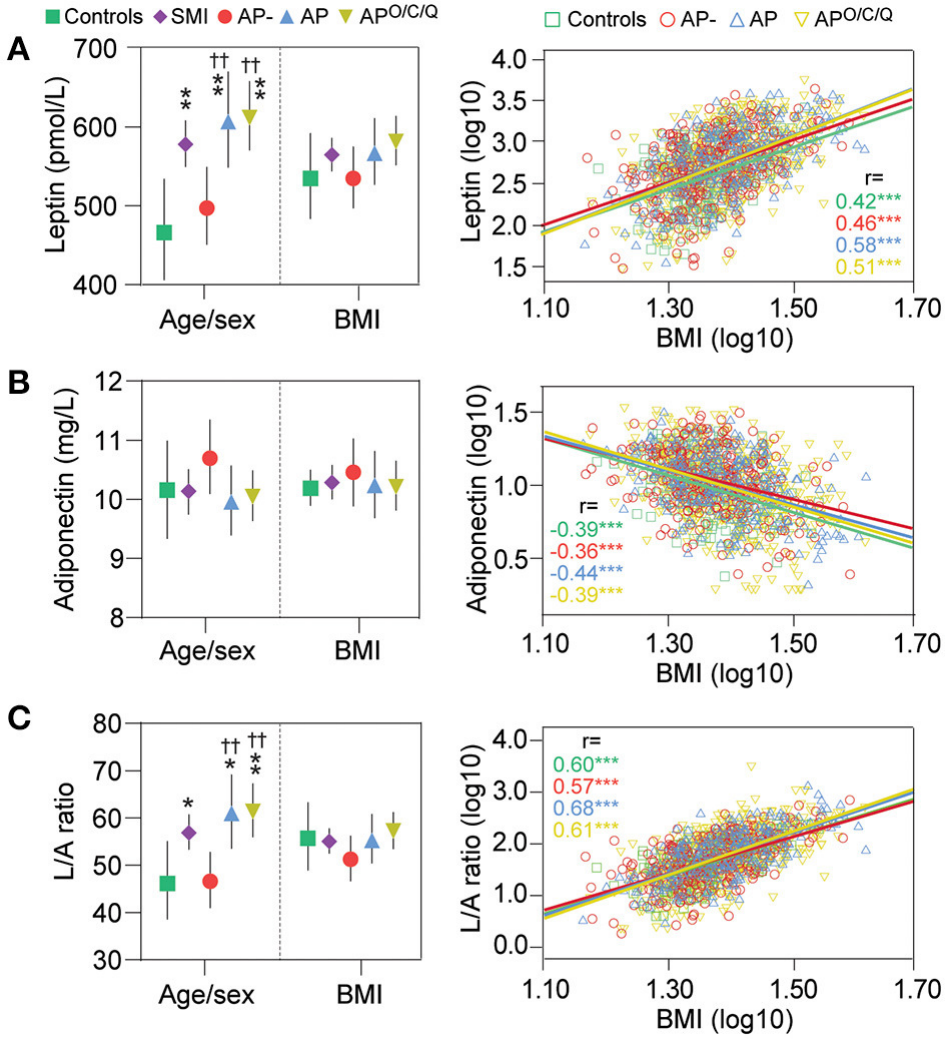
Leptin, adiponectin and their ratio (L/A) in severe mental illness (SMI) and according to antipsychotic (AP) treatment. To the left: multivariate analysis of covariance with dependent variable: leptin **(A)**, adiponectin **(B)** or L/A **(C)**, with adjustment for age, sex, duration of illness, mood stabilizers (DDD), duration of AP treatment and additional adjustment with body mass index (BMI). *p < 0.05 **p < 0.01 ***p < 0.001 vs. healthy controls; ^†^*p* < 0.05, ^††^*p* < 0.01, ^†††^*p* < 0.001 vs. AP-. To the right: correlation between log-transferred leptin, adiponectin or L/A, and BMI. Numbers indicate the regression coefficient. ***p < 0.001.

As shown in Figure 1A, patients as a whole were characteriz1ed by markedly higher leptin levels compared to HC (*p* = 0.005) in age-, sex-, and duration of illness adjusted analysis. Further, patients using AP (both AP and AP^O/C/Q^), had markedly higher leptin levels compared to patients not using AP (AP-), (*p* < 0.01 for both AP groups vs. AP-) and HC (*p* < 0.01 for both AP groups vs. HC), in analysis with further adjustment for AP treatment duration, and treatment with mood stabilizers (anticonvulsants and lithium). The same pattern of higher leptin levels was observed when evaluating the diagnostic groups SCZ or BD according to AP use (Supplementary Figure 1A).

As shown in Figure 1B and in Supplementary Figure 1B, no significant differences in adiponectin levels was observed between HC and SMI or according to AP use with similar findings within SCZ and BD.

As shown in Figure 1C and in Supplementary Figure 1C, the L/A ratio mirrored leptin levels although the differences between groups were somewhat attenuated. Thus, patients with SMI have an elevated L/A ratio compared to HC (*p* = 0.041), and AP users had a higher ratio than non-AP users (*p* < 0.002 for both AP groups) with a similar pattern, but larger confidence intervals attenuated significance, in diagnostic SCZ and BD groups.

Leptin was correlated with duration of illness (*r* = 0.11, *p* < 0.001) and duration of AP treatment (*r* = 0.18, *p* < 0.001. DDD for anticonvulsive therapy correlated modestly with adiponectin levels (*r* = 0.15, *p* < 0.001) and the L/A ratio (*r* = −0.09, *p* = 0.004). DDD for lithium correlated modestly with leptin levels (*r* = 0.11, *p* < 0.001) and the L/A ratio (*r* = −0.09, *p* = 0.002). No other correlation between adipokine levels DDD for lithium and anticonvulsive therapy or duration of illness or AP treatment were detected.

### Adipokine Distribution by BMI

As shown in Figure 1A, leptin and BMI were strongly positively correlated in all groups (*p* < 0.001) and no interaction between BMI and AP group (i.e., group^*^BMI) was observed. Accordingly, the marked differences observed in age- and sex-adjusted analysis were largely mitigated by BMI adjustment and no differences in leptin levels between SMI, HC or AP, AP- and AP^O/C/Q^ groups were observed after this adjustment.

As shown in Figure 1B, adiponectin correlated negatively with BMI (*p* < 0.001 for all groups), but correction for BMI did not reveal differences between the studied groups.

As shown in Figure 1C, the L/A ratio was strongly positively correlated with BMI (*p* < 0.001 for all groups) and the differences between diagnostic and between sub-groups according to AP use were diminished following BMI adjustment.

### Discriminating Power of Adipokines on Atherogenic Lipid Ratios

**Figure 2:**
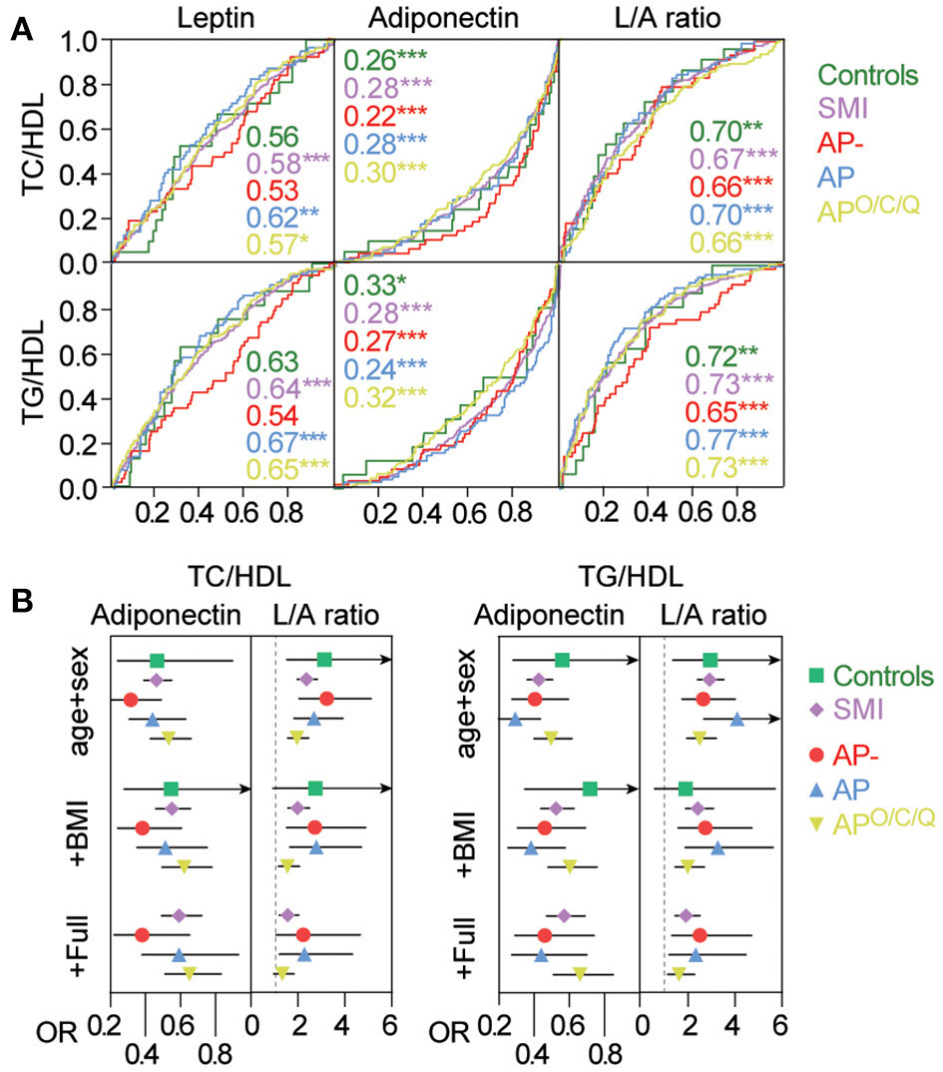
Association between Adiponectin and the L/A ratio and atherogenic risk. **(A)** Association between Adiponectin or the L/A ratio, and having a TC/HDL or TG/HDL above threshold levels as assessed by the area under the receiver-operating characteristics curve (ROC). Numbers indicate the AUC in the different groups. *p < 0.05 **p < 0.01 ***p < 0.001. **(B)** Association between adiponectin and L/A levels and cardiovascular risk (TC/HDL, TG/HDL) using logistic regression with adjustment levels for a. Age and sex b. a+BMI c. b+C-reactive protein (CRP), insulin resistance (HOMA-IR), smoking, and anti-psychotic treatment dose (DDD), duration of illness, mood stabilizers (DDD), duration of AP treatment. Odds ratios (OR) are expressed as log-transformed per SD change in marker. Odds ratios with 95% Confidence interval are given in Supplementary Tables 2, 3.

Figure 2A shows ROC analysis of leptin, adiponectin, and L/A ratio levels ability to discriminate individuals with or without pro-atherogenic lipid ratios above treshold levels. The leptin levels were poor in identifying patients with elevated pro-atherogenic as reflected by the area under the curve (AUC) < 0.7 for TC/HDL and TG/HDL across the groups analyzed. The adiponectin levels showed a fair discrimination of individuals with elevated atherogenic ratios. The reciprocal values of the ROC analysis (i.e., inverse) indicated AUC between 0.7 and 0.8 with no major differences in HC, SMI or according to AP treatment. Notably, the best discrimination was observed in the AP- group with an AUC of 0.78 (inverse of 0.22). The L/A ratio was better than leptin but still poor in identifying individuals with elevated TC/HDL ratio (AUC < 0.7) but somewhat better in identifying individuals with an elevated TG/HDL ratio (AUC > 0.7). This pattern was seen across all groups studied but was notably poorer in the AP- group vs. TG/HDL (AUC = 0.65).

### Association Between Adipokine Levels and Atherogenic Lipid Ratios

We next evaluated the association between adipokine levels and elevated pro-atherogenic ratios using logistic regression with different adjustment levels to assess if the associations were independent or modified by other cardio-metabolic risk factors, mood stabilizers (anticonvulsants and lithium), duration of illness, and duration of AP treatment. Odds ratios (OR) were for these analyses based on log-transformed standardized values, and represent a one standard deviation (SD) increase in the analyzed marker. Based on the poor performance of leptin in the ROC analysis, we focused on adiponectin and the L/A ratio. The results of these analyses are presented in Figure 2B and Supplementary Tables 2, 3. As presented in Figure 2B, the OR for adiponectin and the L/A ratio was largely comparable in age- and sex-adjusted analysis in identifying individuals that had an elevated TC/HDL or TG/HDL ratio with overlapping CI's between all groups. The numeric for this analysis as well as for the diagnostic groups and subgroups according to AP use are presented in Supplementary Tables 2, 3.

The OR's were somewhat attenuated upon addition of BMI but both the L/A ratio and in particular adiponectin, remained significantly associated with an elevated TC/HDL and TG/HDL ratio, also after further adjustment with other cardio-metabolic risk factors (i.e., CRP, smoking, HOMA-IR, DDD for AP), mood stabilizers, duration of illness, and duration of AP treatment to the models. The lack of significance in the HC population is largely due the lower frequency of dyslipidemia in this group as reflected by large CI's but comparable point estimates (OR) were observed in HC compared to the other groups. Altogether, the L/A ratio and in particular adiponectin were independently associated with atherogenic risk in all diagnostic groups and treatment modalities.

## Discussion

We investigated dysregulation of the prototypical adipokines leptin, adiponectin, and leptin/adiponectin ratio in a large clinical sample of patients with SMI, associations with elevated atherogenic lipid ratios, and whether these associations were dependent on antipsychotic treatment. We found that (i) patients with SMI displayed markedly elevated leptin levels and L/A ratio, with particularly high levels observed in AP users while adiponectin was comparable in all groups, that (ii) all markers correlated strongly with BMI with similar associations in all diagnostic groups and AP treatment regimes, and that (iii) low adiponectin and a high L/A ratio were associated with elevated lipid ratios, and (iv) the association with elevated lipid ratios was independent of other cardio-metabolic risk factors; BMI, CRP, insulin resistance and smoking, with comparable associations with risk according to AP use. Altogether, elevated leptin in SMI was largely driven by BMI and not strongly associated with elevated lipid ratios. In contrast, adiponectin was not dysregulated, however, low levels were strongly and independently associated with the elevated lipid ratios. We were unable to detect that any adverse effect of AP treatment on elevated lipid ratios is conveyed by these adipokines as reflected by their circulating levels.

Obesity and overweight are frequent in SMI and are associated with increased leptin levels, closely correlated with BMI and AP treatment, as confirmed in the present study and shown previously in numerous other studies (14, 24). Increased fat mass is accompanied by infiltration of various myeloid immune cells (such as neutrophils, monocytes and macrophages) in adipose tissue, and altered secretion of adipokines including reduced expression of the insulin sensitizing adiponectin (25–27). Furthermore, AP treatment has been shown to promote monocyte infiltration, macrophage effector functions and inflammation in adipose tissue in experimental studies (28–30), as well as to regulate adiponectin expression and secretion (30). Thus, dysregulated adiponectin levels could be expected to be particularly low in AP treatment users. However, in line with recent meta-analyses, adiponectin levels were similar in SMI and HC (31, 32). Except for somewhat higher adiponectin levels in AP-, we did not find the levels to be significantly modified by AP use. While several studies report low levels of adiponectin with AP use, and in particular in patients receiving second generation AP treatment (30, 32), few of these studies compare with HC which in our study showed similar levels as in SMI. The differences in L/A ratio between SMI and HC in our study largely reflect leptin levels, since no major dysregulation in adiponectin was detected. Importantly, leptin and adiponectin were closely correlated with BMI in all diagnostic and treatment groups, with comparable regression coefficients and no interactions between group and BMI, suggesting similar production of these adipokines per unit change in BMI. This argues against a more inflamed adipose tissue in SMI, where augmented or antagonized secretion of leptin and adiponectin would be expected, although regional fat distribution would be more informative in this regard.

Obesity in patients with SCZ and BD may contribute to elevated CVD risk, which may be further augmented by AP treatment use (1). Indeed, atherogenic lipid ratios were markedly enhanced as previously shown (18), especially in SCZ and in AP users in our study. Leptin and adiponectin could contribute to enhanced CVD risk through metabolic pathways related to obesity and insulin resistance, but could have independent effects as well. Adiponectin has been shown to directly affect HDL and TG metabolism, independent of fat mass, insulin resistance and dyslipidemia (33, 34) in non-psychiatric patients, and may modulate signaling pathways in response to inflammatory stimuli in several cell types (35). Thus, adiponectin seems to diminish the inflammatory response in endothelial cells to mechanical injury (36) and modulated macrophages to acquire an anti-inflammatory phenotype and inhibited foam cell transformation (37). Adiponectin has recently been demonstrated to have two forms with opposing actions in endothelial cells (7). However, in line with a protective anti-inflammatory role, we found an inverse association between adiponectin and atherogenic lipid ratios in SMI patients. Furthermore, the association with lipid ratios was markedly stronger than for leptin, and persisted following full adjustment for important cardio-metabolic factors.

Focusing on non-psychiatric patients with metabolic disturbances, numerous clinical studies have shown that dysregulated leptin and adiponectin levels (38, 39) are strongly associated with dyslipidemia. However, as in the present study, studies on these adipokines and CVD are often cross-sectional using surrogate endpoints for CVD such as lipid ratios (40) or coronary artery calcium (41). There are fewer prospective studies evaluating the association between these adipokines and incident CVD or CVD related outcomes in patients with metabolic distrubances. Shanker et al. demonstrated that low adiponectin and high leptin were associated with incident events in patients with coronary artery disease (42). Low adiponectin was associated with future coronary heart disease in type 2 diabetes (43) as well as CV mortality (44). In contrast, high adiponectin was associated with cardiovascular events in patients with hypertension (45), and in older adults (46). Thus, the association between these adipokines and incident CVD or outcomes seem to depend on the degree of metabolic disturbances.

Evaluating diagnostic groups revealed overall stronger associations in BD compared to SCZ. Thus, despite quite similar levels, a unit decrease of adiponectin in BD was associated with a larger risk of having an atherogenic lipid profile than SCZ possibly indicating a more anti-atherogenic effect of adiponectin in BD. As we measured total adiponectin, evaluation of high molecular weight (HMW) adiponectin could have given different results (47). Possibly, different distribution of sub-fractions could also explain the stronger association with CVD risk in BD but we were unable to find any studies evaluating this. Adipose tissue has been shown to sense and respond to emotional stress through peroxisome-profilator activated receptor γ (PPARγ)-adiponectin interactions (48), that are also linked to immunometabolic regulation and systemic inflammation (49), but future studies are needed to evaluate how or if this is relevant in BD.

Increased risk of adverse cardiac events has been associated with AP use, and in particular second generation AP including olanzapine, clozapine, and quetiapine (50). However, we did not observe any clear differences in pro-atherogenic lipid ratios associated with low adiponectin or high L/A ratio, that were dependent on AP use. We found a lower risk of having elevated lipid ratios per unit decrease in adiponectin in users of these 3 s generation AP, arguing against any adverse effects of these drugs acting through the adiponectin signaling pathway, at least as reflected by circulating levels in a cross-sectional setting. Adverse effects of AP could still be mediated by other pathways, both inflammatory [e.g., activated leukocytes in adipose tissue enhancing inflammation (4)], and non-inflammatory [e.g., effects on lipogenesis and lipolysis (51)].

## Limitations

Since fasting status effects the levels of adipokines and other confounding cardiovascular variables, a non-fasting status was an exclusion criteria for this study, thus excluding 520 non-fasting available healthy controls, limiting the size of the HC population. This gave different group sizes of HC vs. SMI. We also excluded 60 non-fasting BD and 158 non-fasting SCZ patients. The patients with a lower function level can find it challenging to fast over night. Many participants, both controls and patients, have busy everyday lives and were not able to schedule blood sampling in the morning. The non-fasting participants are included in other studies in the overall TOP-study.

For the HC in our study smoking status is not available. Although the effect of smoking on inflammation is less documented, the effect of smoking on CVD risk is known. Therefore, since we had smoking status in our patients, we were able to adjust for smoking when evaluating the association between adiponectin and L/A ratio and CVD risk in logistic regression analysis.

Patients with major depression with psychotic symptoms were not included in this study due to small sample size and thus limited statistical power.

We were unable to investigate the possible effect of lipid lowering medication (statins) on CVD risk as only 15 patients in our sample were using these agents. Finally, the design of this study is cross-sectional, limiting our ability to conclude on causality of the associations shown.

Furthermore, as our study is associative by nature, cause–effect relationships would have to be shown in a prospective controlled study.

## Strengths and Clinical Implications

To our knowledge, this is to date the largest study evaluating dysregulation of leptin, adiponectin, and leptin/adiponectin ratio in patients with SMI. The patient population was well-characterized allowing us to adjust our regression models with relevant cardio-metabolic risk factors as well as use of other mood stabilizers and duration of both illness and AP treatment.

As lifespans in people with SMI are markedly reduced frequently due to CVD, there is a need for treatment options that target modifiable metabolic risk factors. Circulating adiponectin represents a modifiable risk factor that can be efficiently targeted by lifestyle modifications, mainly weight loss and dietary changes (52). Thus, low adiponectin could be used to identify and monitor patients that could benefit from such modifications as well as in increasing awareness of increased CVD risk in these individuals.

## Conclusion

In a large clinical sample of patients with SMI we show that adiponectin is not dysregulated in patients compared to HC, but low levels of adiponectin are associated with enhanced CVD risk regardless of AP treatment regime. Our findings support an anti-atherogenic role for adiponectin and suggest it could be further evaluated in novel CVD risk prediction strategies in SMI.

## Data Availability Statement

The datasets presented in this article are not readily available because sharing of data to external parties has not been approved by the ethics committee. Requests to access the datasets should be directed to e.j.reponen@medisin.uio.no.

## Ethics Statement

The studies involving human participants were reviewed and approved by Regional committees for medical and health research ethics, East Norway (REK 1). The patients/participants provided their written informed consent to participate in this study.

## Author Contributions

ER and TU contributed to data collection, literature search, study design, statistical analysis, and manuscript editing. MT contributed to data collection, literature search, statistical analysis, and manuscript editing. ID and TV contributed to data collection, literature search, and manuscript editing. NS, MW, SL, IJ, LR, and OA contributed to data collection and manuscript editing. AS contributed to literature search and manuscript editing. All authors contributed to the article and approved the submitted version.

## Conflict of Interest

OA has received speaker's honorarium from Lundbeck and is a consultant for HealhLytix. The remaining authors declare that the research was conducted in the absence of any commercial or financial relationships that could be construed as a potential conflict of interest.
