# Supplementary material for: Adiponectin Is Related to Cardiovascular Risk in Severe Mental Illness Independent of Antipsychotic Treatment
Source: Front Psychiatry. 2021 May 28;12:623192. doi: 10.3389/fpsyt.2021.623192 (PMC8192708; doi:10.3389/fpsyt.2021.623192)
Supplement: Supplementary file 1 [file Data_Sheet_1.docx]

**Supplemetal Table 1.** Demographics of the study population

| Clinical parameters | HC  (n = 992) | SCZ | BD | Post hoc |
| --- | --- | --- | --- | --- |
| N | 176 | 701 | 391 |  |
| Sex (male) | 113 (64) | 409 (58) | 167 (43) | HC,SCZ>BD |
| Age | 32±8 | 30±10 | 34±12 | BD>SCZ |
| Ethnicity (European) | 173 (98) | 550 (79) | 343 (88) | HC>BD>SCZ |
| Duration of illness, years | N/A | 8.4 (7.8) | 12.5 (10.1) | BD>SCZ |
| Smoking status (daily use) | N/A | 330 (48) | 162 (42) |  |
| Statin use | 0 (0) | 11 (1.6) | 4 (1.3) |  |
| Antipsychotic treatment (DDD) | N/A | 1.11 (1.01) | 0.45 (0.69) | SCZ>BD |
| Duration of AP treatment, months | N/A | 11.6 (24.7) | 6.3 (24.1) | SCZ>BD |
| Anticonvulsants (DDD) | N/A | 0.07 (0.25) | 0.26 (0.45) | BD>SCZ |
| Lithium (DDD) | N/A | 0.02 (0.15) | 0.19 (0.45) | BD>SCZ |
| Cardiometabolic risk factors |  |  |  |  |
| HOMA-IR | 1.2±0.7 | 1.8±1.0 | 1.5±0.8 | SCZ> BD>HC |
| CRP (mg/L) | 1.6±2.2 | 2.4±2.8 | 2.2±2.8 | SCZ,BD>HC |
| BMI | 24.2±3.6 | 26.1±4.9 | 25.3±4.1 | SCZ>HC |
| HDL-c(mmol/L) | 1.47±0.39 | 1.34±0.42 | 1.46±0.45 | HC,BD>SCZ |
| LDL-c (mmol/L) | 2.94±0.88 | 3.18±0.96 | 3.04±0.92 | SCZ>HC |
| Total-c (mmol/L) | 4.70±0.95 | 5.11±1.07 | 5.01±1.07 | SCZ,BD>HC |
| Triglycerides(mmol/L) | 1.07±0.78 | 1.43±0.98 | 1.30±0.99 | SCZ,BD>HC |
| Total-c/HDL-c | 21 (11.9) | 186 (26.5) | 71 (18.3) | SCZ>HC,BD |
| Triglycerides/HDL-c | 17 (9.7) | 210 (30.0) | 91 (23.3) | SCZ,BD>HC |
| Symptom scores |  |  |  |  |
| PANSS total | N/A | 0.24±0.13 | 0.19±0.11 | SCZ>BD |
| CDSS total | N/A | 5.5±4.8 | 4.9±4.6 | SCZ>BD |

Analysed with ANOVA for continuous variables and chi-square test for categorical variables. HC= healthy controls, SCZ= schizophrenia spectrum ,BD= bipolar spectrum, n=number, DDD= defined daily dose, HOMA-IR= homeostasis model assessment for insulin resistance, CRP= C-reactive protein, BMI= body mass index, HDL-c=high density lipoprotein cholesterol, LDL-c= low density lipoprotein cholesterol, Total-c= total cholesterol, mg/L= milligrams per liter, mmol/L= millimoles per liter, PANSS=Positive and Negative Syndrome Scale, CDSS= Calgary Depression Scale for Schizophrenia, N/A= not applicable, n.s.= not significant, *p<0.05 **p<0.01 ***p<0.001 vs. HC.

**Supplemental Table 2.**

|  |  |  | HC | SMI | SCZ | BD |
| --- | --- | --- | --- | --- | --- | --- |
| Adiponectin | TC/HDL | Age,sex | 0.46 (0.24-0.90) 0.022 | 0.46 (0.39-0.55) <0.001 | 0.51 (0.42-0.62) <0.001 | 0.34 (0.24-0.48) <0.001 |
|  |  | +BMI | 0.55 (0.28-1.07) 0.08 | 0.55 (0.46-0.66) <0.001 | 0.62 (0.50-0.76) <0.001 | 0.39 (0.27-0.56) <0.001 |
|  |  | +all | N/A | 0.59 (0.49-0.72) <0.001 | 0.69 (0.55-0.86) 0.001 | 0.37 (0.24-0.56) <0.001 |
|  | TG/HDL | Age,sex | 0.57 (0.29-1.13) 0.11 | 0.44 (0.37-0.51) <0.001 | 0.45 (0.37-0.55) <0.001 | 0.39 (0.29-0.53) <0.001 |
|  |  | +BMI | 0.73 (0.35-1.49) 0.39 | 0.53 (0.45-0.64) <0.001 | 0.57 (0.46-0.70) <0.001 | 0.46 (0.33-0.64) <0.001 |
|  |  | +all | N/A | 0.58 (0.48-0.70) <0.001 | 0.65 (0.52-0.83) <0.001 | 0.45 (0.31-0.66) <0.001 |
| L/A ratio | TC/HDL | Age,sex | 3.14 (1.53-6.44) 0.002 | 2.36 (1.98-2.83) <0.001 | 2.27 (1.84-2.79) <0.001 | 2.59 (1.81-3.70) <0.001 |
|  |  | +BMI | 2.76 (0.94-8.06) 0.064 | 1.98 (1.58-2.49) <0.001 | 1.81 (1.38-2.38) <0.001 | 2.31 (1.48-3.61) <0.001 |
|  |  | +all | N/A | 1.57 (1.21-2.04) 0.001 | 1.38 (1.01-1.89) 0.041 | 2.19 (1.31-3.67) 0.003 |
|  | TG/HDL | Age,sex | 3.03 (1.42-6.44) 0.004 | 2.99 (2.48-3.6) <0.001 | 2.89 (2.32-3.6) <0.001 | 3.21 (2.25-4.56) <0.001 |
|  |  | +BMI | 1.97 (0.65-6.00) 0.23 | 2.50 (1.98-3.16) <0.001 | 2.29 (1.73-3.04) <0.001 | 2.90 (1.88-4.48) <0.001 |
|  |  | +all | N/A | 1.98 (1.52-2.59) <0.001 | 1.66 (1.20-2.28) 0.002 | 2.85 (1.74-4.68) <0.001 |

Logistic regression showing adiponectin and the L/A ratio and prediction of CVD risk (TC/HDL and TG/HDL above threshold levels) in healthy controls (HC), schizophrenia (SCZ), bipolar disorder (BD), and SMI (SCZ+BD), with different adjustment levels. Multivariable adjustment included age, sex, BMI, all: C-reactive protein (CRP), insulin resistance (HOMA-IR), smoking, and anti-psychotic treatment dose (DDD), duration of illness, mood stabilizers (DDD), duration of AP treatment.

**Supplemental Table 3.**

|  |  |  | AP- | AP | APO/C/Q |
| --- | --- | --- | --- | --- | --- |
| Adiponectin | TC/HDL | Age,sex | 0.32 (0.20-0.49) <0.001 | 0.44 (0.31-0.63) <0.001 | 0.53 (0.43-0.66) <0.001 |
|  |  | +BMI | 0.38 (0.24-0.60) <0.001 | 0.51 (0.35-0.75) 0.001 | 0.62 (0.49-0.78) <0.001 |
|  |  | +all | 0.38 (0.22-0.65) 0.001 | 0.59 (0.36-0.93) 0.023 | 0.65 (0.51-0.83) <0.001 |
|  | TG/HDL | Age,sex | 0.41 (0.28-0.60) <0.001 | 0.30 (0.21-0.44) <0.001 | 0.51 (0.41-0.62) <0.001 |
|  |  | +BMI | 0.47 (0.31-0.70) <0.001 | 0.39 (0.26-0.59) <0.001 | 0.61 (0.49-0.77) <0.001 |
|  |  | +all | 0.47 (0.30-0.75) 0.002 | 0.45 (0.28-0.71) 0.001 | 0.67 (0.52-0.86) 0.002 |
| L/A ratio | TC/HDL | Age,sex | 3.24 (2.05-5.12) <0.001 | 2.68 (1.83-3.93) <0.001 | 1.96 (1.56-2.46) <0.001 |
|  |  | +BMI | 2.72 (1.51-4.90) 0.001 | 2.79 (1.65-4.72) <0.001 | 1.55 (1.16-2.06) 0.003 |
|  |  | +all | 2.23 (1.07-4.66) 0.33 | 2.28 (1.21-4.33) 0.011 | 1.33 (0.96-1.983) 0.084 |
|  | TG/HDL | Age,sex | 2.73 (1.82-4.09) <0.001 | 4.18 (2.75-6.37) <0.001 | 2.58 (2.02-3.29) <0.001 |
|  |  | +BMI | 2.82 (1.65-4.81) <0.001 | 3.35 (1.96-5.71) <0.001 | 2.06 (1.53-2.78) <0.001 |
|  |  | +all | 2.58 (1.39-4.479) 0.003 | 2.41 (1.27-4.55) 0.007 | 1.69 (1.21-2.36) 0.002 |

Logistic regression showing adiponectin and the L/A ratio and prediction of CVD risk (TC/HDL and TG/HDL above threshold levels) in patients with severe mental illness according to treatment with antipsychotics: AP-,=patients not using antipsychotic treatment, APO/C/Q= patients using olanzapine, clozapine or quetiapine, AP= patients using other AP, with different adjustment levels. Multivariable adjustment included age, sex, BMI, all: C-reactive protein (CRP), insulin resistance (HOMA-IR), smoking, and anti-psychotic treatment dose (DDD), duration of illness, mood stabilizers (DDD), duration of AP treatment.


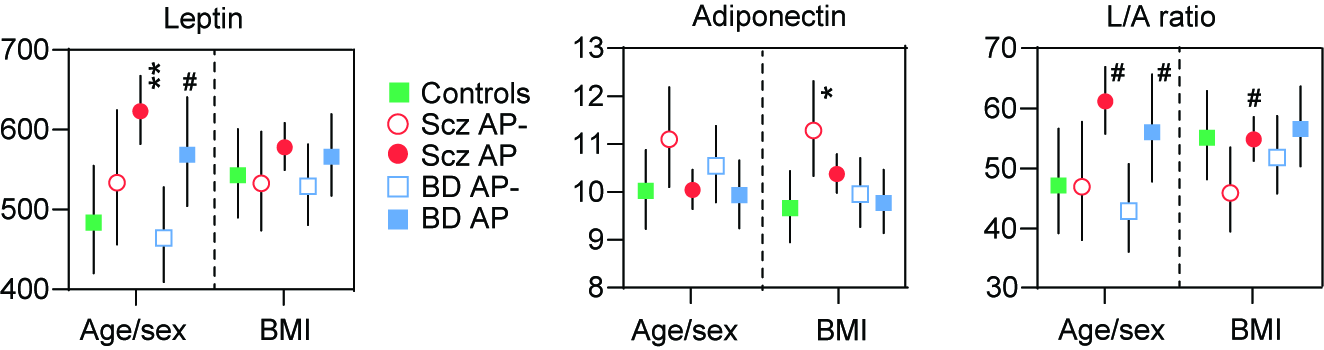


**Supplemental Figure 1. Leptin, adiponectin and their ratio (L/A) in diagnostic groups according to antipsychotic (AP) treatment.** Multivariate analysis of covariance evaluating healthy controls and the diagnostic groups SCZ or BD according to AP use (all categories of AP). Dependent variable: leptin (left), adiponectin (middle) or L/A (right), with adjustment for age and sex, duration of illness, mood stabilizers (DDD), duration of AP treatment and additional adjustment with body mass index (BMI). *p<0.05 **p<0.01 ***p<0.001 *vs.* healthy controls, #p<0.01 SCZ AP vs. SCZ No AP.
